# Editorial: Analysis of innovations in food development: improving nutritional value, flavor and texture in food products

**DOI:** 10.3389/fnut.2026.1788205

**Published:** 2026-02-06

**Authors:** José M. Alvarez-Suarez, Ivan Luzardo-Ocampo

**Affiliations:** 1 Laboratorio de Investigación en Ingeniería en Alimentos (LabInAli), Departamento de Ingeniería en Alimentos, Colegio de Ciencias e Ingenierías, Universidad San Francisco de Quito (USFQ), Quito, Ecuador; 2 Laboratorio de Bioexploración, Colegio de Ciencias Biológicas y Ambientales, Universidad San Francisco de Quito (USFQ), Quito, Ecuador; 3 Tecnologico de Monterrey, Institute for Obesity Research, Monterrey, NL, Mexico; 4 Tecnologico de Monterrey, School of Engineering and Sciences, Monterrey, NL, Mexico

**Keywords:** food innovation, food processing, functional ingredients, nutritional quality, sensory properties, sustainable food systems

The global food industry is undergoing a profound transformation, driven by increasing consumer demand for healthier, more sustainable, and sensorially appealing food products, while simultaneously facing pressing challenges related to food security, nutritional deficiencies, environmental impact, and the rising prevalence of diet-related non-communicable diseases. These converging pressures have positioned innovation in food development not merely as a technological opportunity, but as a strategic necessity for building resilient, nutritious, and socially inclusive food systems. Within this context, improving nutritional value, flavor, and texture has become a central objective in contemporary food science and technology, as these attributes jointly determine both health outcomes and consumer acceptance.

**Figure 1:**
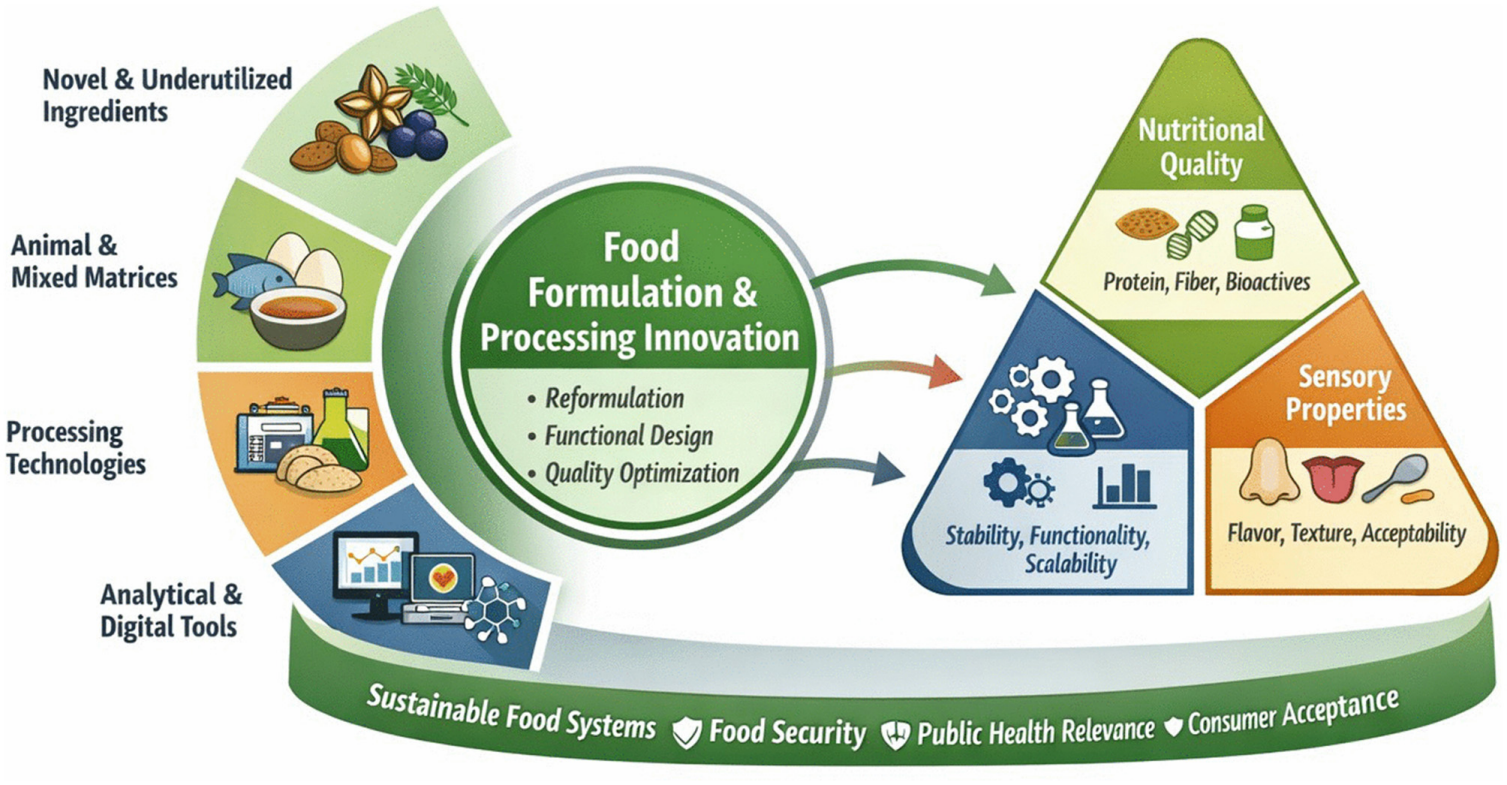
Conceptual framework illustrating the main innovation pathways addressed in this Research Topic. The figure integrates novel ingredients, processing technologies, formulation strategies, and analytical tools as key drivers influencing nutritional quality, sensory properties, and technological performance of food products, ultimately contributing to sustainability, food security, and consumer acceptance.

The Research Topic “*Analysis of Innovations in Food Development: Improving Nutritional Value, Flavor and Texture in Food Products*” was conceived to address these interconnected challenges by bringing together original contributions that explore novel ingredients, innovative processing technologies, formulation strategies, and emerging analytical approaches. The 10 articles included in this Research Topic provide an integrated and interdisciplinary overview of how food innovation can enhance nutritional quality while preserving or improving sensory properties, offering viable alternatives aligned with sustainability and food security goals. Collectively, these studies demonstrate that meaningful progress in food development requires coordinated advances across the entire food value chain, from raw material selection to processing, formulation, evaluation, and consumption. The main innovation pathways and their interconnections across ingredients, processing, formulation, analytical approaches, and quality outcomes addressed in this Research Topic are summarized in Figure 1.

A central topic emerging from several contributions is the valorization of novel, native, and underutilized ingredients as a pathway toward nutritional enhancement and sustainability. Amazonian oilseeds, traditionally exploited primarily for lipid extraction, have recently attracted interest for their broader nutritional and functional potential within circular food systems. While Sacha inchi (*Plukenetia volubilis* L.) oil is widely recognized for its high content of essential fatty acids, comparatively little attention has been given to the nutritional and techno-functional properties of its defatted by-products. To address this gap, Alejandro Ruiz et al. provide a comprehensive characterization of Sacha inchi seeds, oil, and oilcake, demonstrating that the latter is a protein-, fiber-, mineral-, and bioactive-rich ingredient with functional properties suitable for food formulation. Similarly, fruit-derived oils such as açaí have long been valued for their health-related attributes, yet varietal differences in their nutritional and technological quality remain insufficiently explored. In this context, Santos et al. compare oils obtained from purple and white açaí (*Euterpe oleracea* Mart) varieties, revealing distinct fatty acid profiles and nutritional indices with potential cardioprotective implications. Together, these studies underscore the relevance of regional bioresources and ingredient diversity in designing nutritionally superior and sustainable food products.

Ingredient diversification as a strategy for nutritional improvement is not limited to plant-based systems and can be effectively extended to animal-derived and mixed food matrices. Bakery products such as wheat-based burger buns are widely consumed but often low in protein quality and essential amino acid content. Although marine-derived proteins have been proposed as alternative nutrient sources, their incorporation into bakery matrices remains technologically challenging due to potential impacts on texture and acceptability. Within this framework, Ali and El-Anany demonstrate that partial substitution of wheat flour with steamed squid (*Loligo forbesii*) powder significantly improves protein content and essential amino acid profiles while maintaining high consumer acceptability at optimized inclusion levels. In parallel, the growing demand for convenient, nutrient-dense foods has stimulated interest in compact snack formats that deliver both energy and functional benefits. Responding to this need, Alsuhebani et al. develop high-protein energy balls formulated with date paste enriched with Samh seed powder and different dairy protein sources, showing high protein digestibility, antioxidant capacity, and low glycemic response, thereby reinforcing the importance of ingredient synergy in functional food design tailored to modern lifestyles.

Beyond ingredient selection, food processing plays a decisive role in shaping both nutritional and sensory quality, particularly in functional foods derived from traditional food–medicine systems. *Cistanche deserticola*, a plant with long-standing use as a food–medicine homolog, is commonly processed to enhance its functional attributes; however, the influence of modern drying strategies on its quality has remained insufficiently defined. In this context, Jiang et al. systematically evaluate different drying methods applied to fresh and rice wine–steamed *Cistanche*, demonstrating how processing choices directly influence bioactive compound retention, texture, and sensory perception. This contribution illustrates how aligning traditional processing practices with advanced analytical and sensory tools can unlock new value in functional plant-based ingredients.

Flavor and texture, often decisive determinants of consumer acceptance, require precise analytical tools to guide product innovation and quality control. Traditional foods such as peanut porridge are particularly sensitive to raw material quality and processing history, yet the mechanisms underlying flavor differentiation are not fully understood. Addressing this challenge, Yuan et al. employ gas chromatography–ion mobility spectrometry combined with electronic nose and electronic tongue systems to elucidate flavor differences between peanut porridges prepared from aged and fresh peanuts, identifying characteristic volatile compounds and linking chemical profiles to sensory perception. This study advances understanding of flavor formation mechanisms and highlights the utility of intelligent sensory technologies in formulation optimization and product standardization.

Importantly, innovation in food development must also respond to population-level nutritional needs and food security constraints, particularly in low-income settings. Complementary foods play a critical role when breastfeeding alone is insufficient to meet infant nutritional requirements, yet many commercial formulations remain economically inaccessible. In this context, Girma Shewa et al. develop nutrient-rich complementary foods using locally sourced ingredients in Eastern Ethiopia, demonstrating that simple processing techniques, such as germination and roasting, can reduce antinutritional factors while delivering meaningful contributions to infant nutrient intake. This work exemplifies how food innovation can be context-specific, affordable, and socially impactful.

At the system level, improving nutritional quality also requires tools capable of assessing and monitoring large and complex food supplies. Although carbohydrates represent a major source of dietary energy worldwide, information on free sugar content in packaged foods is often limited. To address this gap, Scuccimarra et al. introduce a machine-learning–based approach to predict free sugar content and assess carbohydrate quality across a global database of packaged foods, thereby providing a scalable tool to support reformulation strategies, public health policies, and evidence-based decision-making.

The translational relevance of food innovation is further strengthened when supported by human intervention data. Staple foods such as bread are prime targets for reformulation due to their widespread consumption and metabolic impact. In this regard, Marak et al. demonstrate that long-term consumption of quinoa (*Chenopodium quinoa* W.) flour-supplemented bread significantly improves glycemic index and lipid profiles without compromising product acceptability, reinforcing the role of traditional and ancient crops in modern dietary strategies aimed at improving metabolic health.

Finally, innovation in food systems can also occur upstream through feed-to-food strategies that influence the nutritional quality of animal-derived products. While feed efficiency and cost remain major concerns in poultry production, dietary strategies that enhance final product quality are of increasing interest. Addressing this aspect, Martinez et al. show that supplementation of laying hen diets with *Moringa oleifera* leaves improves egg quality parameters and commercial value without compromising production performance, illustrating how nutritional innovation at the feed level can generate cascading benefits from animal nutrition to human consumption.

In summary, the studies included in this Research Topic provide a substantial and coherent body of evidence demonstrating how innovation in food development—through novel ingredients, processing strategies, intelligent sensory tools, and data-driven approaches—can simultaneously improve nutritional value, flavor, and texture while addressing broader challenges related to sustainability and food security. Together, these contributions highlight viable pathways to developing healthier and more appealing food products that meet both consumer expectations and public health needs. At the same time, it should be acknowledged that food innovation may also involve nutritional, sensory, or technological trade-offs, particularly when introducing novel ingredients or reformulating traditional products, which warrants careful evaluation from a holistic perspective.

The 10 articles presented in this Research Topic, while offering valuable insights, do not cover all dimensions of innovation in food development. As the field continues to evolve, further robust and interdisciplinary research efforts are needed to deepen understanding and expand the evidence base supporting science-driven food innovation.

Future research in this area may be particularly directed toward the following aspects:

a. Integrated assessment of nutritional quality, sensory perception, and technological performance of innovative and reformulated food products, including long-term health implications and consumer acceptance across different cultural contexts.
b. Stability, bioaccessibility, bioavailability, and functional performance of bioactive food components throughout processing, storage, and digestion, and their contribution to product quality and health-related outcomes.
c. Valorization of regional, native, and underutilized food resources as sustainable ingredients for the development of novel food products with enhanced nutritional value and optimized texture and flavor.
d. Application of emerging technologies, including intelligent sensory systems, data-driven modeling, and advanced processing techniques, to support precision formulation, reformulation strategies, and scalable innovation within modern food systems.

Overall, this Research Topic consolidates current evidence demonstrating that targeted innovation in ingredients, processing, formulation, and analytical evaluation can effectively address the interconnected challenges of nutritional quality, sensory performance, and sustainability in food development.

